# Morphological characterization of the progenies of pure and reciprocal crosses of *Pangasianodon hypophthalmus* (Sauvage, 1878) and *Clarias gariepinus* (Burchell, 1822)

**DOI:** 10.1038/s41598-018-22149-4

**Published:** 2018-02-28

**Authors:** Tosin Victor Okomoda, Ivan Chu Chong Koh, Anuar Hassan, Thumronk Amornsakun, Sherif Md Shahreza

**Affiliations:** 1 Department of Fisheries and Aquaculture, College of Forestry and Fisheries, University of Agriculture Makurdi, P. M. B. 2373 Makurdi, Nigeria; 2 School of Fisheries and Aquaculture Sciences, Universiti Malaysia Terengganu, 21030 Kuala Nerus, Terengganu, Malaysia; 3 Department of Technology and Industries, Faculty of Science and Technology, Prince of Songkla University, Pattani campus Thailand, 94000 Mucang Puttani, Thailand; 4 Institute of Tropical Aquaculture, Universiti Malaysia Terengganu, 21030 Kuala Nerus, Terengganu, Malaysia

## Abstract

Twenty-five traditional and thirty-four geometric morphometric comparisons were carried out on pure and reciprocal crosses of *Pangasianodon hypophthalmus* (Sauvage, 1878) and *Clarias gariepinus* (Burchell, 1822). Thirty fish samples each of the *C. gariepinus* (CH), *P. hypophthalmus* (PH), Pangapinus (♀PH × ♂CG) and the two distinct morphotypes of the Clariothalmus (♀CG × ♂PH) (Clarias-like and Panga-like) between the ages of four and six months were used for this study. Phenotypically, the Clarias-like Clariothalmus and the Pangapinus progenies were indistinguishable from their maternal parents while the Panga-like Clariothalmus was a phenotypic intermediary of the putative parents but looks more closely to the paternal parent. Hence, both univariate proportion and multivariate analysis of the collected data successfully separated the different fishes into three multivariate spaces. The analysis of the dendrogram with complete linkage and Euclidean distance further showed the close relationship of the isolated Panga-like Clariothalmus progenies to the paternal parent, however, Clarias-like Clariothalmus and the Pangapinus were completely intermingled with their maternal parents. The most important index of discrimination of these fishes into different multivariate spaces was the fin characteristic which showed 100% exclusive ranges for the individual groups in many cases.

## Introduction

The need for genetic improvement and aquaculture diversification has been the justification for hybridization in fishes. It is one of the biotechnological breeding tools used to develop new genetic stocks of fish for aquaculture/fisheries industries and a viable alternative to selective breeding when there is little additive genetic variation in the desired traits of pure stocks to be exploited^1^. However, successful genetic improvement through hybridization and other biotechnology tools requires proper identification and classification of species^2^. Hybrid identification is a problem linked to natural resources management because fertile hybrids could interbreed with pure species leading to genetic introgression. Despite the reliability of genetic approach, morphological characterization is still widely exploited due to its rapidity and the ease of field applicability^3,4^.

Traditional methods for morphological identification are based on principal component analysis and discriminant function analysis of linear measurements^5^. Discriminant function analysis of morphological data can be used to separate two or more groups of fish individuals into multivariate spaces. However, many authors have elucidated the fact that the result from the traditional morphological measurement is sometimes contradictory and ambiguous^6–8^. Geometric morphometric on the other hand is a landmark-based technique and considered the most rigorous morphometric technique ever^9–11^. It is capable of processing morphometric data from digital images with landmark points quickly and with high precision^9,12,13^. The integration of geometric morphometric data with other analytic tools such as biochemical, geographical, molecular and morphological parameters could better describe phylogenetic relationships among fishes and shed light to many ambiguous taxonomic ranks^14–16^.

Recently, we produced novel hybrid progenies from intergeneric crosses of Asian catfish *Pangasianodon hypophthalmus* (S.) and African catfish *Clarias gariepinus* (B.)^17,18^. Beyond the production of a novel aquaculture candidate, we also anticipated providing solutions to some of the breeding problems associated with the production of the pure crosses by the hybridization between the two species. For instance, the killing of male *C. gariepinus* to obtain testis is perfectly complimented and could be avoided due to the ease of sperm stripping from the male *P. hypophthalmus*. Also, the early maturity (9months) and high fecundity of the female *C. gariepinus* eliminate seasonal production characteristics associated with *P. hypophthalmus* brood fish due to the difficulty in obtaining gravid female (late maturity period of about 3 years). More so, early report of the hybrid ♀*C. gariepinus* × ♂*P. hypophthalmus* has demonstrated superior growth performance and heterosis over both pure parents^17^. In view of the performance characteristics of the hybrids and popularity of the pure crosses, it is important to urgently provide a quick and rapid identification tools for the hybrid since data on genetic discrimination is not available. In this study, morphological data were collected using traditional and geometric measurement hence, overcoming the drawbacks inherent in the use of traditional multivariate techniques alone. It is believed that the combination of both approaches would allow for accurate characterization of the novel hybrids between the African and Asian catfishes.

## Materials and Methods

Progenies of *C. gariepinus* (CH), *P. hypophthalmus* (PH), and the reciprocal crosses Pangapinus (♀PH × ♂CG) and Clariothalmus (♀CG × ♂PH) were obtained from similar breeding history using the method described by Okomoda *et al*.^17,18^. In brief, six sexually mature *P. hypophthalmus* and *C. gariepinus* (between 1–2.5 kg) were injected with Ovaprim^®^ hormone at a dosage of 0.5 ml/kg. After stripping of the females, the pooled eggs of the different species were divided separately into two portions. One portion was used for the production of pure progenies (♀CG × ♂CG and ♀PH × ♂PH); while the other portion was used for the reciprocal crosses Clariothalmus (♀CG × ♂PH) and Pangapinus (♀PH × ♂CG).

**Figure 1:**
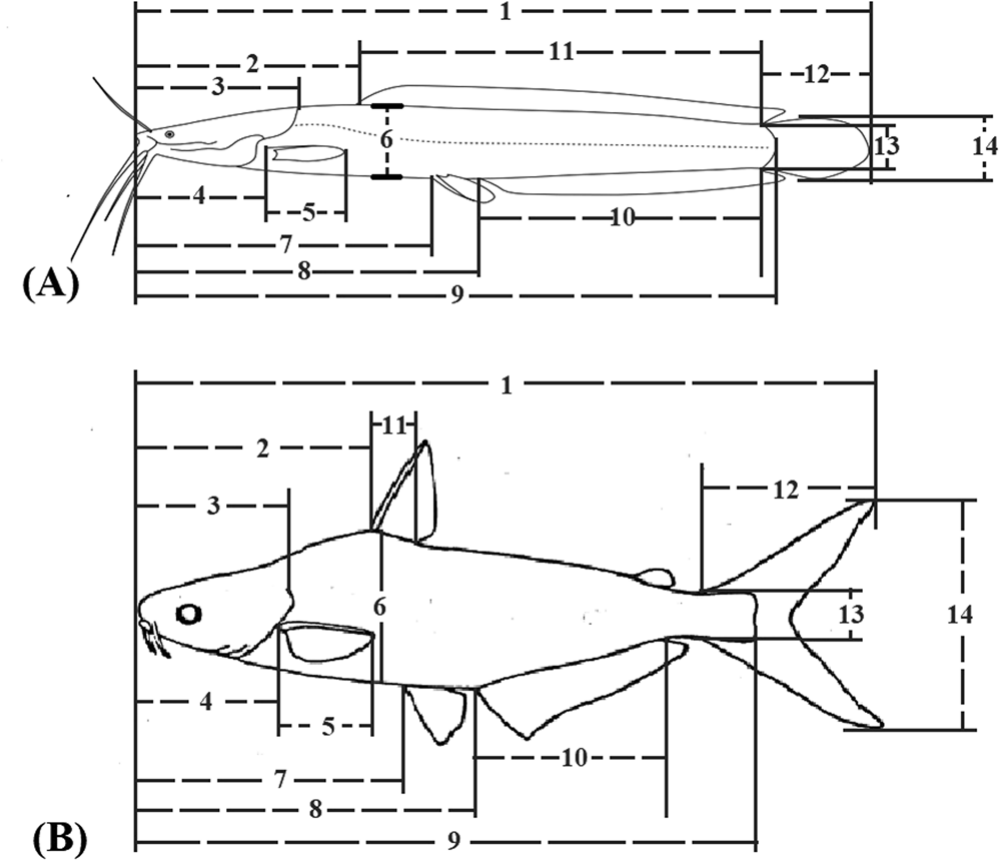
Some traditional morphometric measurement for (**A**) *Clarias gariepinus* (**B**) *Pangasianodon hypophthalmus*. 1 = Total length; 2 = Pre-dorsal distance; 3 = head length; 4 = pre-pelvic distance; 5 = pelvic height; 6 = body height; 7 = pre-pectoral distance; 8 = pre-anal distance; 9 = standard length; 10 = Anal fin length; 11 = Dorsal fin length; 12 = Caudal fin height; 13 = Caudal peduncle depth; 14 = Caudal fin length.

The progenies obtained were cultured for at least four months (4–6 months) at the School of Fisheries and Aquaculture Sciences hatchery of the Universiti Malaysia Terengganu, Malaysia before morphological analysis was done. Thirty^19^ fish samples each of the progenies of the pure *C. gariepinus, P. hypophthalmus*, Pangapinus (Panga-like) and the two observed morphotypes of the Clariothalmus (Clarias-like and Panga-like) were used for morphological characterization in this study. The experimental protocols for this study were approved by the Universiti Malaysia Terengganu committee on research. All methods used in this study involving the care and use of animals were in accordance with international, national, and institutional guidelines. Twenty-five conventional traditional morphometric data were collected from each fish (some of which are described in Fig. 1).

These includes total length (TL), standard length (SL), dorsal fin length (DFL), dorsal fin height (DFH), distances between dorsal fin end and adipose fin origin (DBDAF), predorsal distance (PDD), pelvic fin length (PFL), pelvic fin height (PFH), pre pelvic distance (PPD), pectoral fin length (PeFL), pectoral fin height (PeFH), anal fin length (AFL), anal fin height (AFH), caudal fin length (CFL), caudal fin height (CFH), caudal peduncle depth (CPD), body height (BH), body width (BW), head length (HL), head width (HW), eye diameter (ED), upper maxillary barbel length (UMBL), lower maxillary barbel length (LMBL), mouth width (MW) and pre-orbital length (POL).

Body related measurements were expressed as percentages of standard length, while head related parameters were expressed as percentages of head length. Descriptive statistics of data were done and further subjected to analysis of variance using Minitab 14 software. The percentages and exclusive ranges of the data from the fins characters were also determined. This was done by first sorting data in ascending order using the Microsoft Excel software and the exclusive ranges of a paired combination of the different groups were determined. The paired combination evaluated includes; pure *C. gariepinus* vs *P. hypophthalmus*; Clarias-like Clariothalmus vs pure *C. gariepinus*; Clarias-like Clariothalmus vs pure *P. hypophthalmus*; Panga-like Clariothalmus vs pure *C. gariepinus*; Panga-like Clariothalmus vs pure *P. hypophthalmus*; Pangapinus vs pure *C. gariepinus*; Pangapinus vs pure *P. hypophthalmus*; Clarias-like Clariothalmus vs Panga-like Clariothalmus; Clarias-like Clariothalmus vs Pangapinus and Panga-like Clariothalmus vs Pangapinus.

**Figure 2:**
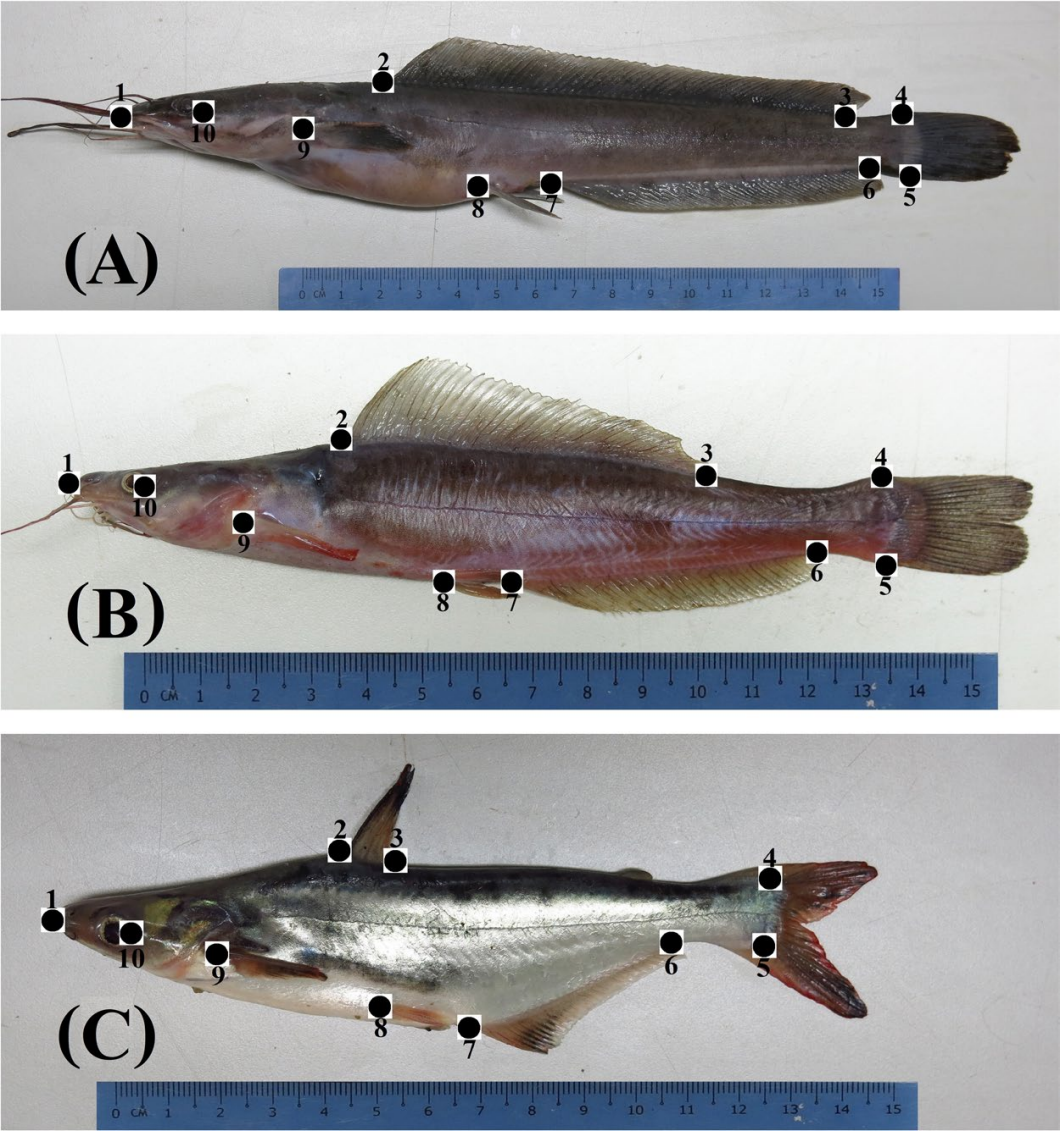
Morphological landmarks points for (**A**) typical pure *Clarias gariepinus*/Clarias-like Clariothalmus (**B**) Panga-like Clariothalmus (**C**) typical Pangapinus/*Pangasianodon hypophthalmus*. Landmark points are; Snout^1^, origin of dorsal fin^2^, posterior end of the dorsal fin^3^, dorsal attachment of the caudal fin to the tail^4^, ventral attachment of the caudal fin to the tail^5^, posterior end of the anal fin^6^, origin of the anal fin^7^, origin of the pelvic fin^8^, origin of the pectoral fin^9^ and the posterior point of the eye^10^.

Trust network method was also used to measure morphological traits. Ten landmark point (Fig. 2) were identified namely Snout^1^, origin of dorsal fin^2^, posterior end of the dorsal fin^3^, dorsal attachment of the caudal fin to the tail^4^, ventral attachment of the caudal fin to the tail^5^, posterior end of the anal fin^6^, origin of the anal fin^7^, origin of the pelvic fin^8^, origin of the pectoral fin^9^ and the posterior point of the eye^10^. Thirty-six distances between the different landmark points were recorded as shown in Table 1. Values from the landmark distance measured were expressed as percentages of standard length.

**Table 1:** Morphometric distances measured between landmarks points of pure and reciprocal *Pangasianodon hypophthalmus* and *Clarias gariepinus*. (Landmark points are as indicated in Fig. 2).

SN	Landmark points	Character description
1		Total length (Snout to the median of the posterior end of the caudal fin)
2		Standard length (Snout to the median of the attachment of the caudal fin to the tail)
3	1–2	Snout to the origin of dorsal fin.
4	2–3	Dorsal fin length.
5	3–4	Posterior end of the dorsal fin to the dorsal attachment of the caudal fin to the tail.
6	4–5	Dorsal attachment of the caudal fin to the tail to ventral attachment of the caudal fin.
7	5–6	Ventral attachment of the caudal fin to the tail to the posterior end of the anal fin.
8	6-7	Anal fin length.
9	7–8	Origin of the anal fin to the origin of the pelvic fin.
10	8–9	Origin of the pelvic fin to the origin of the pectoral fin.
11	9–10	Origin of the pectoral fin to the posterior point of the eye.
12	1–10	Snout to the posterior point of the eye.
13	2–10	Origin of the dorsal fin to the posterior point of the eye.
14	3–10	Posterior end of the dorsal fin to the posterior point of the eye.
15	4–10	Dorsal attachment of the caudal fin to the tail to the posterior point of the eye.
16	8–10	Origin of the pelvic fin to the posterior point of the eye.
17	7-10	Origin of the anal fin to the posterior point of the eye.
18	6–10	Posterior end of the anal fin to the posterior point of the eye.
19	5–10	Ventral attachment of the caudal fin to the tail to the posterior point of the eye.
20	2–9	Origin of the dorsal fin to the origin of the pectoral fin.
21	3–9	Posterior end of the dorsal fin to the origin of the pectoral fin.
22	4–9	Dorsal attachment of the caudal fin to the tail to the origin of the pectoral fin.
23	7–9	Origin of the anal fin to the origin of the pectoral fin.
24	6–9	Posterior end of the anal fin to the origin of the pectoral fin.
25	5–9	Ventral attachment of the caudal fin to the tail to the origin of the pectoral fin.
26	2-8	Origin of the dorsal fin to the origin of the pelvic fin.
27	2–7	Origin of the dorsal fin to the origin of the anal fin.
28	2–6	Origin of the dorsal fin to the posterior end of the anal fin.
29	2–5	Origin of the dorsal fin to the ventral attachment of the caudal fin to the tail.
30	3–8	Posterior end of the dorsal fin to the origin of the pelvic fin.
31	3–7	Posterior end of the dorsal fin to the origin of the anal fin.
32	3–6	Posterior end of the dorsal fin to the posterior end of the anal fin.
33	3–5	Posterior end of the dorsal fin to the ventral attachment of the caudal fin to the tail.
34	4–8	Dorsal attachment of the caudal fin to the tail to the origin of the pelvic fin.
35	4–7	Dorsal attachment of the caudal fin to the tail to the origin of the anal fin.
36	4–6	Dorsal attachment of the caudal fin to the tail to the posterior end of the anal fin.

To ensure that variations in this study were only attributed to body shape differences, and not to the relative sizes of the fish, size effects from the data set were eliminated, by standardizing the morphometric parameters (from traditional and trust network measurement) using the allometric formula given by Elliott *et al*.^20^:

$${{\rm{M}}}_{{\rm{adj}}}={\rm{M}}\,{(\mathrm{Ls}/\mathrm{Lo})}^{{\rm{b}}};$$

where M is the original measurement, M_adj_ is the size-adjusted measurement, Lo is the TL of the fish, and Ls is the overall mean of the TL for all fish from all samples. Parameter b was estimated for each character from the observed data as the slope of the regression of log M on log Lo, using all fish in all groups.

The data collected were subjected to Principal component analysis (PCA) using PAST free software to obtain sample centroids graph and then determine the morphological character that contributes most to the separation of the fishes into distinct groups. Dendrograms with complete linkage and Euclidean distances of the fishes were also determined.

## Result and Discussion

**Figure 3:**
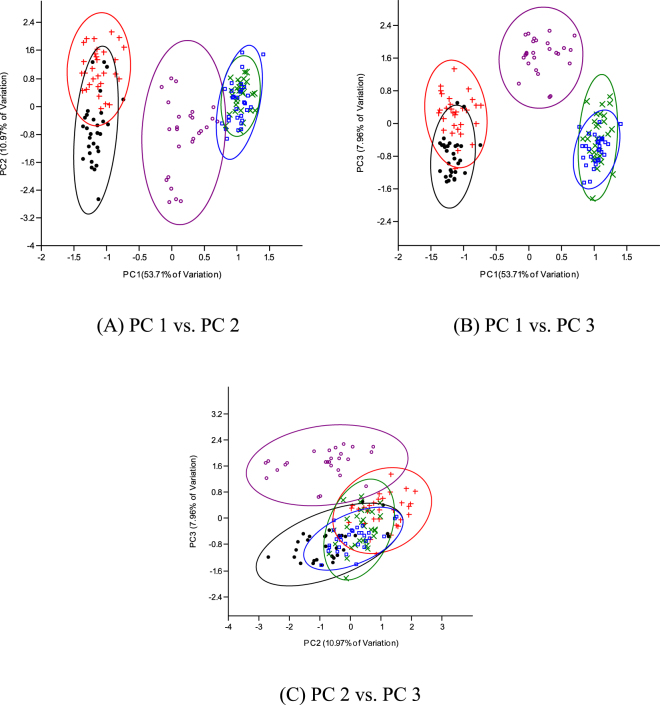
Principal component analysis of transformed morphometric data obtained from pure and reciprocal crosses of *Pangasianodon hypophthalmus* and pure *Clarias gariepinus* (*n* = 30). Cross = pure *C. gariepinus*; dot = Clarias-like Clariothalmus; circle = Panga-like Clariothalmus; multiplication = Pangapinus; square = P.hypophthalmus.

**Figure 4:**
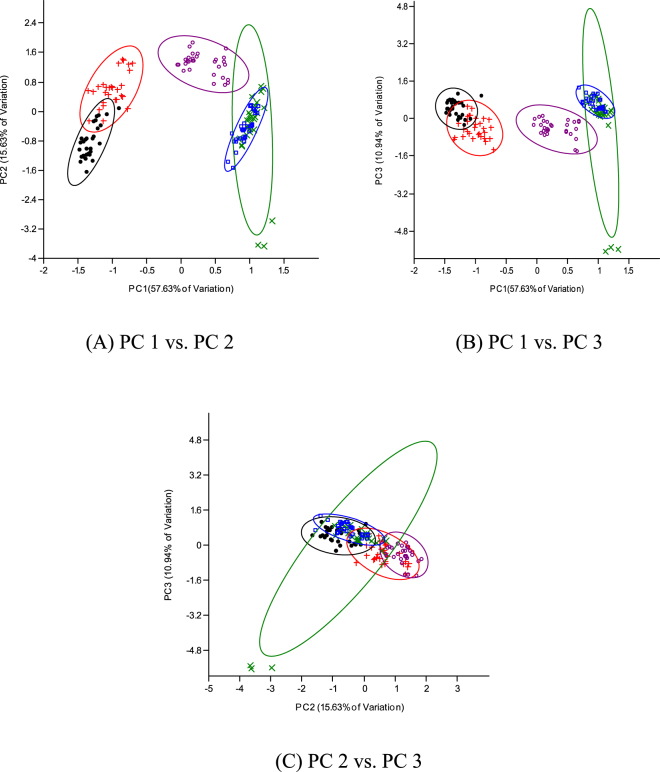
Principal component analysis of transformed morphometric data obtained from pure and reciprocal crosses of *Pangasianodon hypophthalmus* and pure *Clarias gariepinus* (*n* = 30). Cross = pure *C. gariepinus*; dot = Clarias-like Clariothalmus; circle = Panga-like Clariothalmus; multiplication = Pangapinus; square = *P.hypophthalmus*.

**Figure 5:**
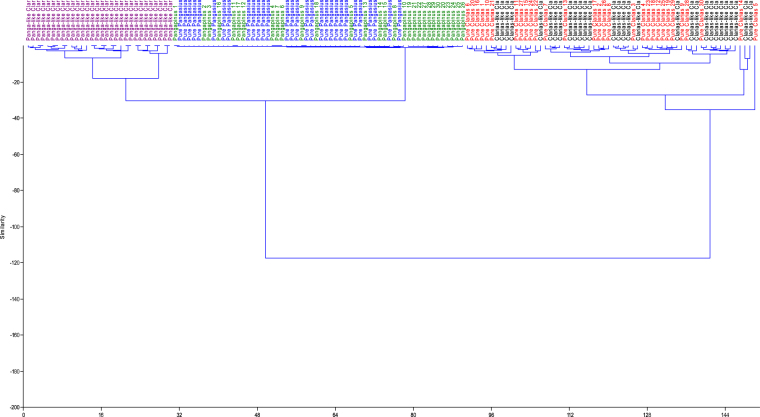
Dendrogram with complete linkage and Euclidean distance for the morphometric parameter of pure and reciprocal crosses of *Pangasianodon hypophthalmus* and pure *Clarias gariepinus* (*n* = 30). Cross = pure *C. gariepinus*; dot = Clarias-like Clariothalmus; circle = Panga-like Clariothalmus; multiplication = Pangapinus; square = *P.hypophthalmus*.

**Figure 6:**
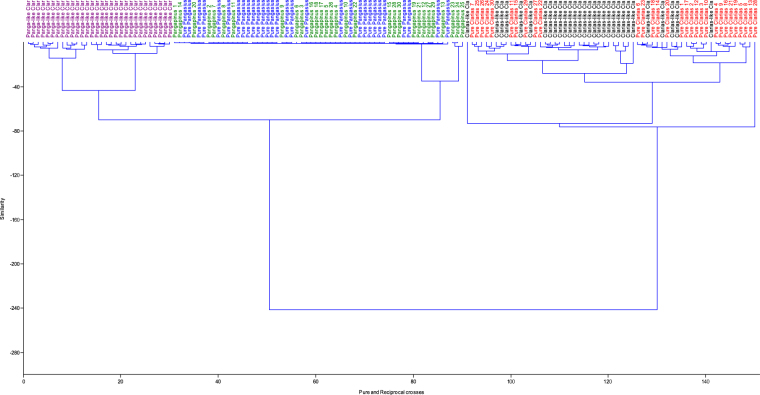
Dendrogram with complete linkage and Euclidean distance for data collected from Trust network on pure and reciprocal crosses of *Pangasianodon hypophthalmus* and pure *Clarias gariepinus* (*n* = 30). Cross = pure *C. gariepinus*; dot = Clarias-like Clariothalmus; circle = Panga-like Clariothalmus; multiplication = Pangapinus; square = *P.hypophthalmus*.

Despite the ease of phenotypic discrimination between two species involved in interspecific or intergeneric crosses, the use of morphological characterization could pose a number of challenges because of inappropriate techniques and the occurrence of growth allometry^16,21^. Hence, discrimination depending solely on size influenced morphometric traits becomes difficult or largely inaccurate. It is, however, important to eliminate size effect or growth-related shape changes and then elucidate shape differences among the different fish groups. The morphometric parameters expressed as a proportion of standard/head length revealed three distinct multivariate spaces for the five fish groups in this study. The Clarias-like Clariothalmus and the Pangapinus progenies were indistinguishable phenotypically to their maternal parents and had similar morphological and geometric proportions (Tables 2–4). The Panga-like Clariothalmus on the other hand had intermediate/shared features of both parents, however, looks more like the paternal parent. This is justified by the discriminant function analysis of morphometric which clustered the Panga-like Clariothalmus between the parental clusters with slight or no overlapping (Tables 5 and 6; Figs 3 and 4) indicating possible intermediate inheritance for the studied characters^1^. Similarly, the analysis of the dendrogram with complete linkage and Euclidean distance showed the closeness of Panga-like Clariothalmus to their paternal parent but uniquely separated (Figs 5 and 6). However, Clarias-like Clariothalmus and the Pangapinus were intermingled with their maternal parents. This is a pointer to differentiation in genetic inheritance in the hybrid progenies of the reciprocal cross.

**Table 2:** Body related traditional morphometric parameters of pure and reciprocal crosses of *Pangasianodon hypophthalmus* and *Clarias gariepinus* expressed as percentages of standard length. Numbers in each cell are means in percentages (%) ± standard error.

Parameter	♀CG × ♂CG	♀CG × ♂PH		♀PH × ♂CG	♀PH × ♂PH	P-Value
		Clarias-like	Panga-like			
DFL	61.89 ± 0.35^a^	62.53 ± 0.30^a^	37.13 ± 0.70^b^	7.13 ± 0.14^c^	6.76 ± 0.09^c^	0.001
DFH	6.35 ± 0.19^c^	5.97 ± 0.12^c^	12.01 ± 0.22^b^	18.37 ± 0.20^a^	17.78 ± 0.21^a^	0.001
DBDAF	—	—	10.71 ± 0.25^b^	32.72 ± 0.25^a^	33.17 ± 0.18^a^	0.001
PDD	31.60 ± 0.31^c^	31.53 ± 0.32^c^	32.78 ± 0.25^b^	35.45 ± 0.27^a^	36.21 ± 0.19^a^	0.001
PFL	2.93 ± 0.08^c^	3.01 ± 0.04^c^	3.24 ± 0.10^b^	4.28 ± 0.11^a^	4.34 ± 0.07^a^	0.001
PFH	9.03 ± 0.15^b^	9.06 ± 0.17^b^	10.44 ± 0.20^a^	10.65 ± 0.17^a^	10.87 ± 0.18^a^	0.001
PPD	45.06 ± 0.42^a^	46.32 ± 0.30^a^	45.34 ± 0.34^a^	43.67 ± 0.24^c^	42.97 ± 0.29^c^	0.001
PeFL	4.58 ± 0.08^c^	4.87 ± 0.09^b^	5.04 ± 0.07^a^	4.76 ± 0.06^bc^	4.52 ± 0.06^c^	0.001
PeFH	11.99 ± 0.19^c^	12.21 ± 0.16^c^	14.27 ± 0.21^b^	15.42 ± 0.13^a^	14.99 ± 0.17^a^	0.001
AFL	42.42 ± 0.36^a^	42.37 ± 0.24^a^	35.68 ± 0.39^b^	31.50 ± 0.30^c^	30.67 ± 0.22^c^	0.001
AFH	4.63 ± 0.13^c^	4.61 ± 0.09c	9.36 ± 0.41^b^	13.29 ± 0.18^a^	12.82 ± 0.13^a^	0.001
CFL	8.65 ± 0.12^c^	8.82 ± 0.11c	10.09 ± 0.18^b^	10.87 ± 0.16^a^	10.50 ± 0.15^a^	0.001
CFH	18.15 ± 0.28^c^	14.97 ± 0.21^d^	20.40 ± 0.19^b^	25.20 ± 0.37^a^	25.42 ± 0.34^a^	0.001
CPD	7.86 ± 0.12^b^	7.78 ± 0.09b	8.44 ± 0.15^a^	8.53 ± 0.09^a^	8.41 ± 0.07^a^	0.001
BH	16.91 ± 0.31^c^	17.07 ± 0.25^c^	20.18 ± 0.52^b^	22.97 ± 0.39^a^	22.66 ± 0.19^a^	0.001
BW	16.98 ± 0.37^a^	17.08 ± 0.29^a^	16.94 ± 0.54^a^	13.54 ± 0.52^c^	13.13 ± 0.17^c^	0.001

Mean in the same row with different superscript differ significantly (P < 0.05).

Keys: DFL = Dorsal fin length; DFH = Dorsal fin height; DBDAF = Distances between dorsal fin end and adipose fin origin; PDD = Pre dorsal distance; PFL = Pelvic fin length; PFH = Pelvic fin height; PPD = Pre pelvic distance; PeFL = Pectoral fin length; PeFH = Pectoral fin height; AFL = Anal fin length; AFH = Anal fin height; CFL = Caudal fin length; CFH = Caudal fin height; CPD = Caudal peduncle depth; BH = Body height, BW = Body width.

**Table 3:** Head related morphometric of pure and reciprocal crosses of *Pangasianodon hypophthalmus* and *Clarias gariepinus* expressed as percentages of head length. Numbers in each cell are means in percentages (%) ± standard error.

Parameter	♀CG × ♂CG	♀CG × ♂PH		♀PH × ♂CG	♀PH ×♂PH	P-Value
		Clarias-like	Panga-like			
HW	67.47 ± 1.75^b^	67.77 ± 0.94^b^	62.47 ± 1.63^b^	73.56 ± 1.76^a^	72.96 ± 0.79^a^	0.001
ED	9.52 ± 0.27^c^	8.03 ± 0.19^c^	12.80 ± 0.25^b^	19.48 ± 0.51^a^	18.86 ± 0.24^a^	0.001
UMBL	101.50 ± 2.80^a^	109.34 ± 2.18^a^	83.82 ± 2.22^b^	55.43 ± 3.47^c^	57.58 ± 1.11^c^	0.001
LMBL	96.76 ± 2.47^a^	95.85 ± 1.60^a^	64.49 ± 2.34^b^	29.67 ± 2.42^c^	29.02 ± 0.62^c^	0.001
MW	39.73 ± 1.11^b^	38.68 ± 0.38^b^	37.13 ± 0.99^b^	45.79 ± 1.35^a^	47.90 ± 1.05^a^	0.001
POL	23.75 ± 0.67^b^	25.12 ± 0.33^b^	24.73 ± 0.27^b^	29.39 ± 0.81^a^	28.63 ± 0.50^a^	0.001

Mean in the same row with different superscript differ significantly (P < 0.05).

Keys: HL = Head length; HW = Head width; ED = Eye diameter; UMBL = Upper maxillary barbel length; LMBL = Lower maxillary barbel length; MW = Mouth width; POL = Pre-orbital length.

**Table 4:** Distance between landmark points expressed as percentages of the standard length of pure and reciprocal crosses of *Pangasianodon hypophthalmus* and *Clarias gariepinus*. Numbers in each cell are means values in percentages (%) ± standard error.

Landmark/SL	♀CG × ♂CG	♀CG × ♂PH		♀PH × ♂CG	♀PH × ♂PH	P-Value
		Clarias-like	Panga-like			
1–2	34.75 ± 0.18^b^	34.94 ± 0.30^b^	34.59 ± 0.30^b^	40.46 ± 0.15^a^	40.38 ± 0.14^a^	0.001
2–3	60.22 ± 0.27^a^	60.75 ± 0.28^a^	39.23 ± 0.81^b^	6.84 ± 0.09^c^	6.86 ± 0.08^c^	0.001
3–4	6.02 ± 0.24^c^	5.55 ± 0.18^c^	25.76 ± 0.73^b^	53.06 ± 0.22^a^	52.58 ± 0.16^a^	0.001
4–5	8.23 ± 0.17^b^	8.97 ± 0.11^b^	10.22 ± 0.11^a^	10.28 ± 0.08^a^	10.44 ± 0.09^a^	0.001
5–6	5.04 ± 0.14^c^	4.92 ± 0.18^c^	9.03 ± 0.20^b^	14.13 ± 0.18^a^	14.26 ± 0.14^a^	0.001
6–7	39.92 ± 0.34^a^	39.58 ± 0.24^a^	34.78 ± 0.37^b^	29.91 ± 0.16^c^	30.33 ± 0.17^c^	0.001
7–8	10.79 ± 0.23^b^	11.41 ± 0.22^b^	10.97 ± 0.29^b^	13.84 ± 0.16^a^	14.20 ± 0.15^a^	0.001
8–9	25.01 ± 0.29^a^	26.28 ± 0.31^a^	23.95 ± 0.20^c^	24.34 ± 0.20^b^	24.11 ± 0.25^b^	0.001
9–10	13.75 ± 0.15^a^	13.48 ± 0.14^a^	12.26 ± 0.18^b^	11.28 ± 0.15^c^	11.65 ± 0.14^c^	0.001
1–10	8.67 ± 0.20^b^	7.90 ± 0.13^b^	10.20 ± 0.18^a^	10.30 ± 0.11^a^	9.95 ± 0.13^a^	0.001
2–10	26.17 ± 0.22^b^	27.44 ± 0.30^b^	25.16 ± 0.26^b^	31.55 ± 0.12^a^	31.79 ± 0.14^a^	0.001
3–10	85.31 ± 0.27^a^	86.90 ± 0.26^a^	63.09 ± 0.70^b^	37.19 ± 0.11^c^	37.40 ± 0.17^c^	0.001
4–10	90.88 ± 0.23^a^	92.23 ± 0.23^a^	88.11 ± 0.26^b^	88.20 ± 0.20^b^	88.19 ± 0.16^b^	0.001
8–10	38.46 ± 0.31^a^	38.95 ± 0.32^a^	35.76 ± 0.26^b^	35.07 ± 0.15^b^	35.01 ± 0.20^b^	0.001
7–10	47.96 ± 0.28^a^	47.76 ± 0.32^a^	45.54 ± 0.20^b^	47.51 ± 0.25^a^	47.76 ± 0.25^a^	0.001
6–10	87.27 ± 0.25^a^	88.41 ± 0.17^a^	79.48 ± 0.28^b^	74.28 ± 0.18^c^	74.34 ± 0.15^c^	0.001
5–10	92.11 ± 0.26^a^	92.95 ± 0.17^a^	87.95 ± 0.24^c^	88.66 ± 0.17^b^	88.87 ± 0.21^b^	0.001
2–9	16.96 ± 0.27^b^	16.92 ± 0.31^b^	16.20 ± 0.36^b^	23.28 ± 0.27^a^	23.23 ± 0.15^a^	0.001
3–9	72.36 ± 0.29^a^	74.24 ± 0.29^a^	51.91 ± 0.60^b^	28.55 ± 0.85^c^	27.77 ± 0.13^c^	0.001
4–9	78.05 ± 0.32^ab^	79.82 ± 0.29^a^	76.79 ± 0.41^c^	77.34 ± 0.22^bc^	77.70 ± 0.18^bc^	0.001
7–9	34.84 ± 0.30^b^	34.70 ± 0.32^b^	33.44 ± 0.31^c^	36.53 ± 0.64^a^	36.53 ± 0.27^a^	0.001
6–9	74.30 ± 0.27^a^	75.90 ± 0.21^a^	67.86 ± 0.32^b^	64.75 ± 0.91^c^	63.64 ± 0.17^c^	0.001
5–9	79.37 ± 0.26^a^	80.04 ± 0.19^a^	76.13 ± 0.36^c^	77.84 ± 0.09^b^	78.08 ± 0.28^b^	0.001
2–8	18.76 ± 0.26^c^	19.77 ± 0.26^c^	20.45 ± 0.34^bc^	22.84 ± 0.53^a^	21.56 ± 0.16^ab^	0.001
2–7	26.92 ± 0.16^b^	27.57 ± 0.23^b^	27.13 ± 0.24^b^	37.82 ± 1.06^a^	39.11 ± 0.19^a^	0.001
2–6	62.89 ± 0.26^a^	63.47 ± 0.26^a^	57.48 ± 0.34^b^	49.51 ± 0.86^c^	47.60 ± 0.20^c^	0.001
2–5	67.83 ± 0.29^a^	67.84 ± 0.27^a^	65.62 ± 0.28^a^	62.27 ± 0.34^b^	62.04 ± 0.18^b^	0.001
3–8	49.54 ± 0.28^a^	50.39 ± 0.31^a^	31.66 ± 0.43^b^	20.65 ± 0.43^c^	19.50 ± 0.17^c^	0.001
3–7	39.32 ± 0.33^a^	39.40 ± 0.23^a^	23.57 ± 0.43^c^	27.26 ± 0.91^b^	25.43 ± 0.17^b^	0.001
3–6	7.18 ± 0.17^c^	7.61 ± 0.13^c^	19.51 ± 0.54^b^	42.72 ± 0.82^a^	40.69 ± 0.20^a^	0.001
3–5	9.95 ± 0.25^c^	10.10 ± 0.18^c^	27.31 ± 0.65^b^	55.67 ± 0.20^a^	55.08 ± 0.22^a^	0.001
4–8	54.98 ± 0.35^b^	55.39 ± 0.30^b^	54.75 ± 0.42^b^	57.13 ± 0.19^a^	57.21 ± 0.18^a^	0.001
4–7	44.61 ± 0.34^c^	44.90 ± 0.24^c^	45.40 ± 0.32^b^	46.27 ± 0.16^a^	46.87 ± 0.18^a^	0.001
4–6	8.18 ± 0.22^c^	9.88 ± 0.13^c^	12.29 ± 0.20^b^	17.02 ± 0.18^a^	17.31 ± 0.11^a^	0.001

Mean in the same row with different superscript differ significantly (P < 0.05). (landmark distance is as stated in Table 1).

**Table 5:** Principal component analysis of transformed morphometric data of the progenies of pure and reciprocal crosses of *Pangasianodon hypophthalmus* and *Clarias gariepinus* (*n* = 30). Numbers are component loading.

Variable	PC1	PC2	PC3	PC4
TL	0.18	−0.01	0.43	−0.10
SL	0.10	−0.08	0.39	0.40
DFL	0.27	0.05	0.07	−0.01
DFH	0.27	0.04	0.003	0.01
DBDAF	−0.12	−0.21	−0.51	0.10
PDD	−0.14	−0.17	0.47	0.11
PFL	−0.23	−0.06	0.22	−0.13
PFH	−0.18	−0.04	0.02	−0.31
PPD	0.17	−0.31	0.07	0.10
PeFL	0.05	−0.36	−0.07	−0.40
PeFH	−0.23	−0.08	0.01	0.01
AFL	0.26	0.06	−0.00	−0.04
AFH	0.26	0.05	0.03	−0.04
CFL	−0.21	−0.20	0.07	0.01
CFH	−0.25	0.13	0.004	−0.08
CPD	−0.16	−0.38	0.07	−0.10
BH	−0.23	−0.19	0.04	−0.09
BW	0.17	−0.27	−0.17	−0.18
HL	0.14	−0.31	−0.17	0.42
HW	0.15	−0.33	0.07	−0.09
ED	−0.26	−0.01	−0.08	0.09
UMBL	0.25	0.004	−0.13	−0.07
LMBL	0.26	−0.03	−0.11	−0.04
MW	0.15	−0.17	0.12	−0.45
POL	−0.02	−0.37	−0.004	0.26
Eigenvalue	13.43	2.74	1.99	1.13
% of variance	53.71	10.97	7.96	4.52
Cumulative % variance	53.71	64.68	72.64	77.16

Keys: TL = Total length; SL = Standard length; DFL = Dorsal fin length; DFH = Dorsal fin height; DBDAF = Distances between dorsal fin end and adipose fin origin; PDD = Pre dorsal distance; PFL = Pelvic fin length; PFH = Pelvic fin height; PPD = Pre pelvic distance; PeFL = Pectoral fin length; PeFH = Pectoral fin height; AFL = Anal fin length; AFH = Anal fin height; CFL = Caudal fin length; CFH = Caudal fin height; CPD = Caudal peduncle depth; BH = Body height, BW = Body width; HL = Head length; HW = Head width; ED = Eye diameter; UMBL = Upper maxillary barbel length; LMBL = Lower maxillary barbel length; MW = Mouth width; POL = Pre−orbital length.

**Table 6:** Principal component analysis of transformed data from landmark point of the progenies of pure and reciprocal crosses of *Pangasianodon hypophthalmus* and *Clarias gariepinus* (n = 30). Numbers are component loading.

Variable	PC1	PC2	PC3	PC4	PC5
Total Length	−0.16	−0.17	0.10	−0.09	−0.04
Standard length	−0.14	−0.23	0.15	−0.10	0.18
1–2	0.14	−0.24	0.12	0.24	−0.15
2–3	−0.22	−0.002	−0.01	−0.02	−0.07
3–4	−0.20	−0.04	−0.03	0.18	−0.21
4–5	0.15	−0.05	0.10	−0.26	0.32
5–6	−0.21	0.05	−0.06	0.05	−0.02
6–7	−0.21	0.04	−0.01	−0.16	−0.02
7–8	0.13	−0.20	0.14	0.18	−0.34
8–9	−0.13	−0.20	0.06	0.17	0.47
9–10	−0.18	0.01	−0.03	0.10	−0.07
1–10	0.16	0.09	−0.04	0.001	0.13
2–10	0.11	−0.29	0.15	0.23	−0.19
3–10	−0.22	0.03	−0.03	−0.01	−0.01
4–10	−0.18	−0.18	0.11	−0.09	0.06
8–10	−0.19	−0.12	−0.01	0.21	0.27
7–10	−0.12	−0.29	0.09	0.27	0.13
6–10	−0.22	−0.03	0.01	−0.03	0.03
5–10	−0.19	−0.18	0.12	−0.05	0.01
2–9	0.16	−0.26	0.08	0.12	−0.08
3–9	−0.20	−0.05	−0.16	−0.08	−0.04
4–9	−0.10	0.07	0.43	0.08	0.09
7–9	0.01	−0.33	−0.28	−0.05	−0.01
6–9	−0.20	−0.11	−0.12	−0.11	0.02
5–9	−0.09	0.10	0.43	0.13	0.06
2–8	0.10	−0.26	−0.18	0.03	0.27
2–7	0.09	−0.31	−0.24	−0.05	0.07
2–6	−0.20	−0.003	−0.15	−0.15	0.07
2–5	−0.14	0.16	0.33	0.04	0.12
3–8	−0.22	−0.01	−0.03	−0.04	−0.10
3–7	−0.19	−0.14	−0.09	−0.07	−0.21
3–6	−0.21	0.03	−0.04	0.08	−0.15
3–5	−0.21	0.02	−0.04	0.08	−0.14
4–8	0.01	−0.24	0.25	−0.35	−0.29
4–7	0.03	−0.17	0.24	−0.54	−0.03
4–6	0.20	−0.11	0.10	−0.10	−0.04
Eigenvalue % of variance	20.39	5.66	3.93	1.71	1.07
	56.63	15.73	10.94	4.77	2.97
Cumulative % variance	56.63	72.36	83.3	88.07	91.04

In interspecific hybridization between Yellow flounder *Limanda ferruginea* (S.) and Winter flounder *Pseudopleuronectes americanus* (W.), morphometric assessed by body proportions indicated that hybrids display a morphology intermediate between the maternal and paternal species^22^. Bhowmick *et al*.^23^ and Jana^24^ had also earlier reported that the ♀ *Gibelion catla* (H.) × ♂ *Labeo rohita* (H.) hybrid exhibited intermediate morphometric traits of the parents. However, this hybrid tends more towards the paternal parent (*L. rohita*) with regard to body proportions and fin ray counts. The coloration of the body of the hybrid was reported similar to *G. catla* while the shape of the mouth was more like those of *L. rohita*. Also, the hybrid ♀*C. gariepinus* × ♂*Clarias batrachus* (L.) was phenotypically similar to *C. batrachus*^25^. The reciprocal hybrid between *Pangasius djambal* (B.) and *P. hypophthalmus* showed intermediate phenotypic characteristics but had a strong similarity with the latter than the former^26^. Legendre *et al*.^27^ and Akinwande *et al*.^2^, had earlier concluded that the display of intermediate phenotypic characteristics in the ♀*C. gariepinus* × ♂*Heterobranchus longifilis* (V.) hybrid was an indication of true hybridization. Hence, it is thought that they resulted from the fusion of the genetic material of both parents. They further hypothesized that phenotypical inheritance in true hybrid has paternal dominance. In the study, the Panga-like Clariothalmus progeny had an adipose fin and a forked tail just like the paternal parent. However, it has an elongated dorsal fin just like those in the maternal parent (but shorter than), hence may be the only true hybrid of the reciprocal crosses. According to Chevassus^28^, fertilization of a fish egg by a heterospecific sperm may lead to the production of haploid, gynogenesis/androgenesis, hybrid diploid, hybrid triploid, hybrid tetraploid, a combination of some or all the mentioned or death. Progenies of many distance hybridization are often composed of different phenotypic characters^29^ which is suggestive of significant differences in genetic composition and inheritance pattern^17^. Therefore, the largely indistinguishable morphology of Purebred *C. gariepinus* and *P. hypophthalmus* with the Clarias-like Clariothalmus and Pangapinus progenies respectively may be a pointer to the presence of gynogenetic individuals. Environmental, geographical, and genetic adaptation has been implicated as possible causes of phenotypic variations between strains in many previous studies^19,30^, observations of the present study, however, is largely linked to differences in species and pattern of genetic inheritance in the different morphotypes of the reciprocal hybrids.

The morphometric variability among the three groups in this study was mainly due to the variation of characters related to fins, and body characteristic. This is because the effect of size was successfully eliminated by the allometric transformation and demonstrated by univariate proportion and multivariate analysis. However, with 100% exclusive ranges observed for the different groups for most fin characters (Tables 7–11), this is likely the easiest and most rapid index of discrimination of the three groups applicable in the field. Haddon and Willis^31^ stated that morphometrics of the head and body depth have been regarded as the most important characters for discrimination of Devil anglerfish *Lophius vomerinus* (V.), Pacific herring *Clupea pallasi pallasi* (V.) and Orange roughy *Hoplostethus atlanticus* (C.)^31^. Solomon *et al*.^19^, however, reported head length, body depth at anus and the eye diameter as the most influential morphometric parameters used to discriminate fish strains from cultured and wild *Clarias gariepinus*. However, the suitability of fin and body characteristics in the study as opposed to reports from the previous study may be due to the clear and unambiguous intergeneric phenotypic differences of the pure crosses used. Based on the results of the present study, the fin characteristic appears to be the most promising index of morphological discrimination. Despite the fact that morphological approach alone is insufficient to investigate hybridization status of fishes, this research has provided useful assumption on the nature of the hybrid progenies and a quick/cheap identification tools for field application. However, a combination of both morphological, genetic and cytogenetic data in future studies could provide a clearer understanding of the nature of the progenies gotten.

**Table 7:** Comparison between Pure *Pangasianodon hypophthalmus* and *Clarias gariepinus* for the exclusive ranges of fin measurement expressed as a percentage of standard length (n = 30). Minimum-maximum exclusive range observed (percentage).

Parameter	Pure *C. gariepinus vs* Pure P. *hypophthalmus*	
	Pure ♀CG × ♂CG	Pure ♀PH × ♂PH
DFL	57.35–67.22 (100%)	5.49–7.65 (100%)
DFH	4.39–9.00 (100%)	14.47–20.47 (100%)
PFL	1.82–3.26 (100%)	3.42–4.92 (100%)
PFH	−(0.0%)	11.69–12.19 (13.33%)
PeFL	5.09–5.39 (20.0%)	3.75–3.85 (3.33%)
PeFH	10.23–12.96 (76.67%)	14.01–16.96 (83.33%)
AFL	37.96–46.06 (100%)	28.48–33.52 (100%)
AFH	3.29–6.45 (100%)	11.10–14.10 (100%)
CFL	6.63–9.00 (100%)	9.43–11.73 (96.67%)
CFH	13.89–20.37 (96.67%)	22.58–30.25 (96.67%)

Keys: DFL = Dorsal fin length; DFH = Dorsal fin height; PFL = Pelvic fin length; PFH = Pelvic fin height; PeFL = Pectoral fin length; PeFH = Pectoral fin height; AFL = Anal fin length; AFH = Anal fin height; CFL = Caudal fin length; CFH = Caudal fin height.

**Table 8:** Exclusive ranges and percentages of fin measurement expressed as a percentage of standard length for Clarias-like Clariothalmus in comparison with the pure parents (n = 30). Minimum-maximum recorded range (percentage).

Parameter	Exclusive range with ♀CG × ♂CG		Exclusive range with ♀PH × ♂PH	
	Pure ♀CG × ♂CG	Clarias-like ♀CG × ♂PH	♀CG × ♂PH	Pure ♀PH × ♂PH
DFL	57.34–59.66 (13.3%)	−(0.0%)	5.49–7.65 (100%)	59.79–66.01 (100%)
DFH	8.06–9.00 (6.67%)	−(0.0%)	14.47–20.47 (100%)	4.65–7.46 (100%)
PFL	1.82–2.53 (60%)	3.32–3.45 (13.3%)	3.42–4.92 (100%)	2.61–3.38 (96.67%)
PFH	11.22–11.68 (10.0%)	7.41–8.50 (30.0%)	11.25–12.19 (50.0%)	7.40–7.53 (3.33%)
PeFL	3.85–3.98 (10.0%)	5.43–5.84 (16.67%)	3.75–3.85 (6.67%)	5.11–5.84 (30.0%)
PeFH	10.23–10.26 (3.33%)	13.97–14.46 (3.33%)	14.47–16.95 (66.67%)	10.26–14.46 (93.33%)
AFL	37.96–39.78 (10.0%)	−(0.0%)	28.48–33.52 (100.0%)	40.0–45.64 (100.0%)
AFH	6.02–6.45 (3.33%)	−(0.0%)	11.45–14.10 (100%)	3.59–6.02 (100%)
CFL	6.63–9.00 (56.67%)	9.09–9.89 (36.67%)	10.00–11.73 (90.0%)	−(0.0%)
CFH	18.28–21.32 (46.67%)	12.69–13.77 (16.67%)	21.13–30.25 (100%)	12.69–18.23 (100%)

Keys: DFL = Dorsal fin length; DFH = Dorsal fin height; PFL = Pelvic fin length; PFH = Pelvic fin height; PeFL = Pectoral fin length; PeFH = Pectoral fin height; AFL = Anal fin length; AFH = Anal fin height; CFL = Caudal fin length; CFH = Caudal fin height.

**Table 9:** Exclusive ranges and percentages of fin measurement expressed as a percentage of standard length for Panga-like Clariothalmus in comparison with the pure parents (n = 30). Minimum-maximum recorded range (percentage).

Parameter	Exclusive range with ♀CG × ♂CG		Exclusive range with ♀PH × ♂PH	
	Purez ♀CG × ♂CG	Panga-like ♀CG × ♂PH	Pure ♀PH × ♂PH	Panga-like ♀CG × ♂PH
DFL	57.35–67.22 (100%)	32.58–44.22 (100%)	5.49–7.65 (100%)	32.58–44.22 (100%)
DFH	4.40–9.00 (100%)	10.07–13.97 (100%)	14.47–20.47 (100%)	10.07–13.97 (100%)
PFL	1.82–1.98 (13.3%)	3.29–4.31 (56.67%)	4.32–4.92 (63.33%)	2.04–3.38 (60%)
PFH	8.52–8.55 (6.67%)	11.81–12.84 (10.0%)	7.53–8.59 (3.33%)	12.19–12.84 (3.33%)
PeFL	3.85–4.06 (13.33%)	5.41–5.59 (20.0%)	3.75–4.03 (13.33%)	5.15–5.59 (53.33%)
PeFH	10.23–13.98 (70.0%)	14.05–17.58 (63.3%)	−(0.0%)	12.80–12.90 (20.0%)
AFL	37.96–46.01 (100%)	32.87–39.46 (100%)	28.48–32.69 (93.3%)	33.60–39.46 (86.67%)
AFH	3.29–5.69 (96.67%)	6.76–15.38 (90.0%)	−(0.0%)	6.08–11.22 (90.0%)
CFL	6.62–7.35 (43.33%)	9.38–11.03 (86.67%)	11.11–11.73 (23.33%)	7.38–7.60 (6.67%)
CFH	13.89–18.81 (66.67%)	21.67–22.29 (16.67%)	22.58–30.25 (96.67%)	18.82–21.11 (83.33%)

Keys: DFL = Dorsal fin length; DFH = Dorsal fin height; PFL = Pelvic fin length; PFH = Pelvic fin height; PeFL = Pectoral fin length; PeFH = Pectoral fin height; AFL = Anal fin length; AFH = Anal fin height; CFL = Caudal fin length; CFH = Caudal fin height.

**Table 10:** Exclusive ranges and percentages of fin measurement expressed as a percentage of standard length for Pangapinus in comparison with the pure parents (n = 30). Minimum-maximum exclusive range observed (percentage).

Parameter	Exclusive range with ♀CG × ♂CG		Exclusive range with ♀PH × ♂PH	
	Pure ♀CG × ♂CG	♀PH × ♂CG Progeny	Pure ♀PH × ♂PH	♀PH × ♂CG Progeny
DFL	57.35–67.22 (100%)	6.02–8.87 (100%)	5.49–6.02 (3%)	8.04–8.87 (13.3%)
DFH	4.39–9.00 (100%)	16.08–20.67 (100%)	14.47–15.83 (6.67%)	20.47–20.67 (3.33%)
PFL	−(0.0%)	3.23–3.26 (3.33%)	−(0.0%)	5.19–5.44 (13.33%)
PFH	8.52–9.72 (50.0%)	11.69–14.39 (36.67%)	7.53–9.64 (13.33%)	12.57–14.39 (16.67%)
PeFL	3.85–3.98 (10.0%)	5.39–5.44 (3.33%)	3.75–4.03 (13.33%)	5.06–5.44 (23.33%)
PeFH	10.23–13.97 (100%)	14.05–16.94 (100%)	13.33–14.01 (20.0%)	−(−)
AFL	10.23–13.97 (100%)	14.05–16.94 (100%)	28.48–28.96 (10.0%)	34.26–36.61 (6.67%)
AFH	3.29–6.45 (100%)	11.56–15.83 (100%)	11.45–11.51 (6.67%)	14.19–15.83 (13.33%)
CFL	6.62–9.00 (100%)	9.63–12.94 (100%)	7.60–9.62 (3.33%)	12.00–12.95 (16.67%)
CFH	13.89–21.32 (100%)	21.97–28.98 (100%)	28.98–30.25 (3.33%)	−(0.0%)

Keys: DFL = Dorsal fin length; DFH = Dorsal fin height; PFL = Pelvic fin length; PFH = Pelvic fin height; PeFL = Pectoral fin length; PeFH = Pectoral fin height; AFL = Anal fin length; AFH = Anal fin height; CFL = Caudal fin length; CFH = Caudal fin height.

**Table 11:** Comparison between the progenies of Clariothalmus and Pangapinus for the exclusive ranges of fin measurement expressed as a percentage of standard length (n = 30). Minimum-maximum exclusive range observed (percentage).

Parameter	Within Clariothalmus			Clariothalmus vs Pangapinus		
	Clarias-like ♀CG × ♂PH	Panga-like ♀CG × ♂PH	Clarias-like ♀CG × ♂PH	♀PH × ♂CG Progeny	Panga-like ♀CG × ♂PH	♀PH × ♂CG Progeny
DFL	59.79–66.01 (100%)	32.58–44.22 (100%)	59.79–66.01 (100%)	6.02–8.87 (100%)	32.58–44.22 (100%)	6.02–8.87 (100%)
DFH	4.65–7.46 (100%)	10.07–13.97 (100%)	4.65–7.46 (100%)	16.08–20.67 (100%)	10.07–13.97 (100%)	16.08–20.67 (100%)
PFL	−(0.0%)	3.49–4.31 (33.33%)	2.61–3.45 (80.0%)	3.51–5.44 (93.33%)	2.04–3.15 (43.33)	4.27–5.44 (46.67%)
PFH	7.41–8.39 (26.67%)	11.49–12.84 (23.33%)	7.41–11.20 (80.0%)	11.27–11.27 (70.0%)	8.59–9.41 (26.67%)	12.93–14.39 (13.33%)
PeFL	5.62–5.84 (10.0%)	−(0%)	5.56–5.84 (13.33%)	−(0%)	5.51–5.59 (13.33%)	4.08–4.05 (3.33%)
PeFH	10.26–12.60 (73.33%)	14.66–17.58 (40.0%)	10.26–14.46 (96.67%)	14.67–16.94 (90.0%)	12.80–14.19 (56.7%)	−(0%)
AFL	40.0–45.64 (100%)	32.87–39.46 (100%)	40.0–45.64 (100%)	29.19–36.61 (100%)	37.07–39.46 (33.33%)	29.19–36.61 (76.67%)
AFH	3.59–6.02 (100%)	11.56–15.83 (100%)	3.59–6.02 (100%)	11.56–15.83 (100%)	6.08–11.22 (90.0%)	15.38–15.83 (2.33%)
CFL	−(0.0%)	9.92–11.03 (80.0%)	7.65–9.60 (96.67%)	10.07–12.95 (86.67%)	7.38–9.38 (20.0%)	11.04–12.95 (30.0%)
CFH	12.69–18.22 (100%)	18.82–22.29 (100%)	12.69–18.23 (100%)	21.96–28.99 (100%)	18.82–21.67 (86.67%)	11.04–12.95 (96.67%)

Keys: DFL = Dorsal fin length; DFH = Dorsal fin height; PFL = Pelvic fin length; PFH = Pelvic fin height; PeFL = Pectoral fin length; PeFH = Pectoral fin height; AFL = Anal fin length; AFH = Anal fin height; CFL = Caudal fin length; CFH = Caudal fin height.
